# Antimicrobial Susceptibility Patterns and Wild-Type MIC Distributions of Anaerobic Bacteria at a German University Hospital: A Five-Year Retrospective Study (2015–2019)

**DOI:** 10.3390/antibiotics9110823

**Published:** 2020-11-18

**Authors:** Mohamed Tarek Badr, Benjamin Blümel, Sandra Baumgartner, Johanna M. A. Komp, Georg Häcker

**Affiliations:** 1 Institute of Medical Microbiology and Hygiene, Medical Center, Faculty of Medicine, University of Freiburg, 79104 Freiburg, Germany; benjamin.bluemel@uniklinik-freiburg.de (B.B.); sandra.baumgartner@uniklinik-freiburg.de (S.B.); johanna.komp@uniklinik-freiburg.de (J.M.A.K.); georg.haecker@uniklinik-freiburg.de (G.H.); 2 IMM-PACT-Program, Faculty of Medicine, University of Freiburg, 79104 Freiburg, Germany; 3 BIOSS Centre for Biological Signaling Studies, University of Freiburg, 79104 Freiburg, Germany

**Keywords:** drug susceptibility testing, anaerobic bacteria, EUCAST, wild-type cutoff value, epidemiologic cut-off value (ECV), ECOFFinder, antibiotic resistance, Germany

## Abstract

Local antimicrobial susceptibility surveys are crucial for optimal empirical therapy guidelines and for aiding in antibiotic stewardship and treatment decisions. For many laboratories, a comprehensive overview of local antimicrobial susceptibility patterns of anaerobic bacteria is still lacking due to the long incubation time and effort involved. The present study investigates the antimicrobial susceptibility patterns and related clinical and demographic data of 2856 clinical isolates of anaerobic bacteria that were submitted for analysis to the Institute for Medical Microbiology and Hygiene of the Freiburg University Medical Center (a tertiary university medical center in Southern Germany) between 2015 and 2019. Antimicrobial susceptibility testing has been carried out according to the European Committee on Antimicrobial Susceptibility Testing (EUCAST) guideline. Minimum inhibitory concentration (MIC)_50_ and MIC_90_ for penicillin, metronidazole, moxifloxacin, and clindamycin were established for Gram-positive anaerobes and for ampicillin-sulbactam, meropenem, metronidazole, moxifloxacin, and clindamycin for Gram-negative anaerobes. The distribution of MIC-values for various antibiotics against anaerobic bacteria was also established, especially for those having no specific breakpoints according to EUCAST guidelines. Most clinically relevant anaerobic bacteria originated from general surgery, neurological, and orthopedic wards. A high proportion of isolates were resistant to moxifloxacin and clindamycin indicating the importance of their susceptibility testing before administration. Based on our study metronidazole and other β-lactam/β-lactamase inhibitor combinations such as ampicillin-sulbactam remain suitable for empirical treatment of infections with anaerobic bacteria.

## 1. Introduction

Anaerobic bacteria constitute an important part of the human microbiome, and they play a substantial role in various human infections, such as central nervous system (CNS), intra-abdominal, and foreign body infections [1,2], especially those of a polymicrobial nature [3]. *Bacteroides*, *Prevotella*, *Cutibacterium* species, and Gram-positive cocci are among the most commonly isolated anaerobic bacteria from clinical samples [3,4]. Previous studies showed variable proportions of isolated anaerobic bacteria and antimicrobial susceptibility depending on infection type and hospital, indicating the importance of local comprehensive studies [5,6,7,8]. Cultivation of anaerobic bacteria is time-consuming and labor-intensive, so culture is often not attempted. Many anaerobic bacteria cannot be cultured at all in standard conditions, and many new species of anaerobic organisms are being discovered through culture-independent approaches such as next-generation sequencing at the present [9,10]. These new approaches have helped us better understand the role played by these organisms in many diseases, and especially their opportunistic nature when taking advantage of host-barrier defects.

Culture of anaerobic bacteria may take several days before antimicrobial susceptibility testing can be carried out, and it takes considerable time before antibiotic therapy can be steered accordingly. In addition, many laboratories do not routinely carry out such costly tests. Many therapy regimens of anaerobic infections are, accordingly, empiric in nature. Knowledge of susceptibility patterns to first-line antibiotics and the clinical origin of anaerobic bacteria are necessary to guide empiric therapy choices appropriately and to contribute to the best possible patient outcome. Various studies have shown noticeable variance in the susceptibility to main antibiotic groups between different anaerobic isolates from different countries and very few of these studies document the relevant clinical characteristics of these patients [8,11,12,13]. In addition, some guidelines such as the European Committee on Antimicrobial Susceptibility Testing (EUCAST) clinical breakpoints lack recommendations for some of the commonly used antibiotics for treatment of anaerobic infections (e.g., moxifloxacin); therefore, interpretation of susceptibility tends to vary between laboratories. In this study we aim to document the trends within the groups of anaerobic bacteria in a university hospital in Southern Germany over the last 5 years, to understand how antibiotic susceptibility and clinical characteristics vary across different species. We aim at defining the MIC distributions of our wild-type (WT) populations to some of the antimicrobial agents lacking clinical breakpoints and estimating local WT cutoff value (COwt) when applicable.

## 2. Results

### 2.1. Characterization of Isolated Anaerobic Bacteria

**Figure 1:**
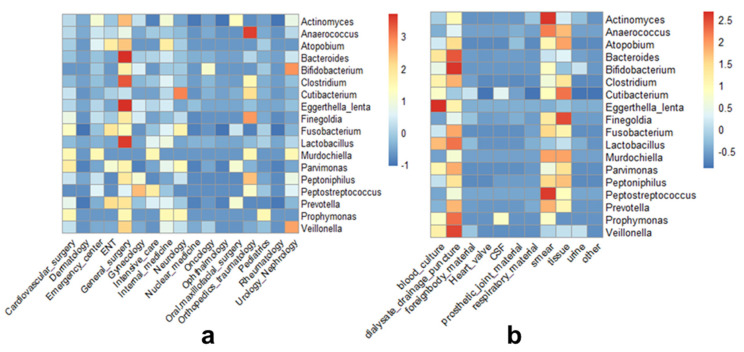
Heatmap of the isolate number of anaerobic bacteria distributed across (**a**) wards or (**b**) samples. The color represents normalized Z-score value where higher scores indicate higher number of isolates.

From January 2015 to December 2019, a total of 2856 isolates of anaerobic bacteria were included in this retrospective study. The majority of isolates were cultured from samples of male patients (61%). For three samples gender was unknown. Swab (n = 647, 22.7%) and tissue (n = 734, 25.7%) specimens were the most common materials. Most samples were submitted by general surgical (n = 528), neurological (n = 454), and orthopedic (n = 433) wards (Supplementary Table S1). Distribution of the different genera of anaerobic bacteria over hospital wards and different sample types are shown in (Figure 1). Anaerobic Gram-positive bacteria represented 80.5% (n = 2298) of all isolates. The most commonly isolated genus among them was *Cutibacterium (Propionibacterium)* (n = 1232) originating mostly from the neurology and orthopedics wards, likely due to their typical role in foreign body infections [14]. Other common genera were *Actinomyces* (n = 331) and *Clostridium* (n = 233). Anaerobic Gram-negative bacteria comprised 19.5% (n = 558) of all isolates. The most frequently genera isolated were *Bacteroides* (n = 253) from which 52.6% were the species *Bacteroides fragilis, Prevotella* (n = 148), and *Fusobacterium* (n = 102).

### 2.2. Antimicrobial Susceptibility Patterns

**Figure 2:**
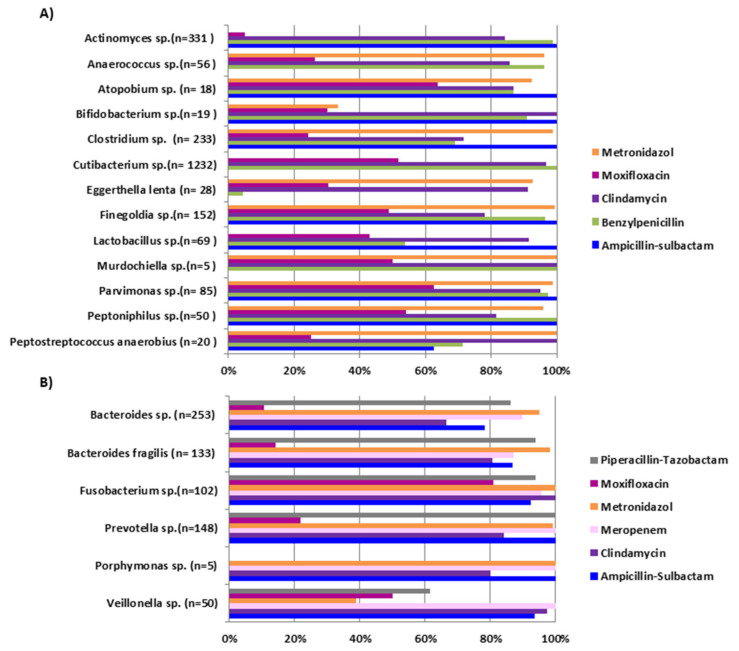
Percentage of susceptibility of selected antibiotics against tested (**A**) Gram-positive and (**B**) Gram-negative anaerobes.

The results of antimicrobial susceptibility testing (percentages of susceptible bacteria; MIC_50_ and MIC_90_) for anaerobic Gram-positive and Gram-negative organisms are shown in Supplementary Tables S2 and S3, respectively. Penicillin resistance showed various patterns of distribution across different isolates. Cumulative percentage of susceptibility for Gram-positive bacilli and cocci was 95.9% and 96%, respectively. *Clostridium*, *Lactobacillus*, and *Eggerthella lenta* isolates showed the greatest number of penicillin resistant phenotypes with only 69%, 53.8%, and 4.3% of the isolates susceptible, respectively. Clindamycin susceptibility varied between Gram-positive and Gram-negative isolates with 91.9% of Gram-positive bacilli and 79% of Gram-negative bacilli being susceptible. Metronidazole showed good activity against most anaerobic isolates both among the Gram-positive (94.5%) and Gram-negative bacilli (97.3%). Highest resistance rates were observed among *Bifidobacterium* (66.7%) and *Veillonella* (61%) isolates. In *Bacteroides* isolates the majority (95%) were susceptible to metronidazole with a MIC_90_ of 4 mg/L. Vancomycin resistance was mainly observed among *Lactobacillus* isolates when compared with other tested Gram-positive bacilli, which is in accordance with data reported by others [15]. Ampicillin-sulbactam showed high susceptibility rates with 100% of Gram-positive bacilli, 92.7% of Gram-positive cocci, and 87.5% of Gram-negative bacilli showing cumulative susceptibility. Percentage of antimicrobial susceptibilities of Gram-positive and Gram-negative anaerobes is shown in Figure 2.

### 2.3. Wild-Type MIC Distributions

**Figure 3:**
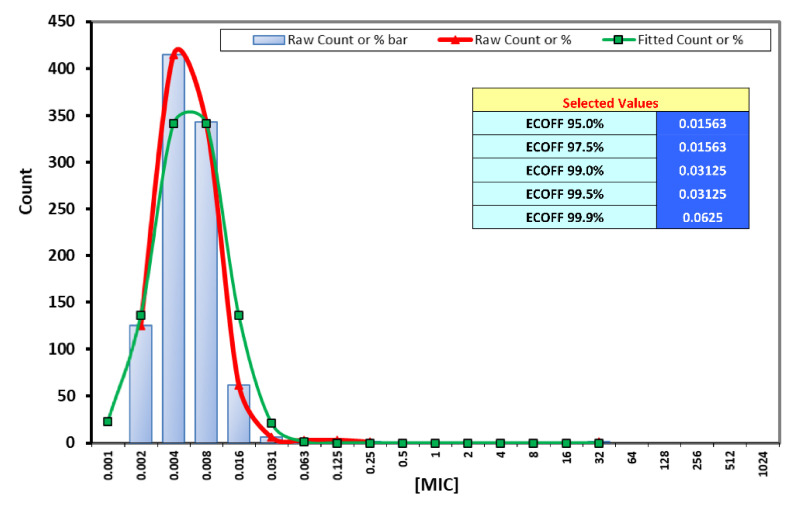
Estimation of wild-type minimum inhibitory concentration (MIC) distributions and ECOFFs for rifampicin against *Cutibacterium.*

Many studies reported the efficacy of rifampicin against anaerobic bacteria, especially in bacterial biofilms and foreign body infections [16]. As there are no breakpoints available for rifampicin and anaerobic bacteria, we investigated the wild-type distribution of rifampicin MICs among the *Cutibacterium* isolates. Most isolates were highly susceptible with MIC_90_ as low as 0.008 mg/L. The ECOFFinder analysis showed that the modal MIC of rifampicin against *Cutibacterium* was 0.003 µg/mL and the 95%, 97.5%, and 99.9% epidemiological cut-off values (ECOFFs) were 0.016, 0.016, and 0.063 µg/mL (Figure 3). We assumed that if the measured MIC of an isolate is in the wild-type distribution then susceptibility of the isolates can be presumed similarly to the ECOFFs defined by EUCAST [17]. Using our wild-type MIC distribution we defined our local WT cutoff value to be the 99.9% ECOFF of 0.0625 and considered isolates with MICs exceeding that cutoff as resistant. With this assumption, 99.4% of the isolates were evaluated as susceptible.

**Figure 4:**
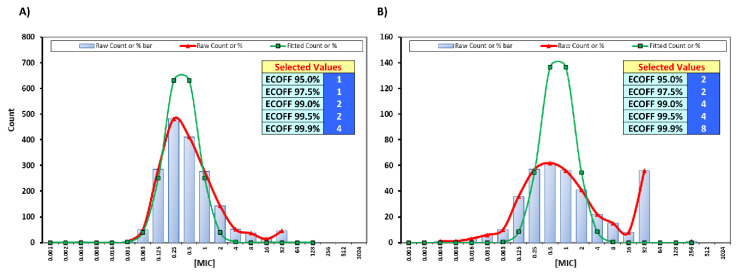
Estimation of wild-type MIC distributions for moxifloxacin against (**A**) Gram-positive and (**B**) Gram-negative bacteria.

Although EUCAST has no defined breakpoints for moxifloxacin against anaerobic bacteria, we have been regularly testing the compound using its EUCAST-defined pharmacokinetic and pharmacodynamic (PK/PD) breakpoints (S ≤ 0.25 mg/L), due to its assumed anaerobic activity [18]. This range lies far below the suggested breakpoint by the Clinical and Laboratory Standards Institute (CLSI) for anaerobic bacteria (S ≤ 2 mg/L). Interestingly, using the EUCAST defined PK/PD breakpoint we found very high resistance among our isolates, as only 45.4% of Gram-positive bacilli, 48.6% of Gram-positive cocci, and 28.9% of Gram-negative bacilli were susceptible to it. To estimate the MIC wild-type distributions of our Gram-positive and negative anaerobic isolates for moxifloxacin we used the ECOFFinder analysis. The modal MICs of moxifloxacin were 0.25 and 0.50 µg/mL for Gram-positive and Gram-negative bacteria, respectively. The 95%, 97.5%, and 99.9% ECOFFs were for Gram-positive and Gram-negative bacteria 1, 1, and 4 µg/mL and 2, 2, and 8 µg/mL respectively (Figure 4). Further MIC distributions for various antibiotics against Gram-positive and negative anaerobes can be found in Supplementary Figure S1.

## 3. Discussion

Despite the increased attention given to anaerobic bacteria in various infections [19], the efforts to decipher their antimicrobial susceptibility patterns across different countries and patient cohorts are still limited to central and reference laboratories in many countries, presumably mainly due to their lengthy and costly cultivation and susceptibility testing. Therefore, large studies are needed to detect new resistance phenotypes and guide empirical therapy regimes. The advances in identification systems such as matrix assisted laser desorption ionization-time of flight mass spectrometry (MALDI-TOF MS) or molecular based sequencing platforms have made it possible not only to discover various, previously neglected anaerobic bacterial communities, but also to identify them in a reasonable time frame and thus to impact clinical decision making and antibiotic stewardship. Due to differences in susceptibility patterns and available breakpoints we divided the different species we tested into two main groups (Gram-positive and Gram-negative) for the analyses. This can be used also as a guide for therapeutic choice based on the results of the Gram staining, e.g., in urgent clinical samples such as blood cultures, where initial antimicrobial choice is usually based on microscopic results. Over five years we have been able to collect data for a large cohort of 2856 clinically relevant isolates, which would allow for a reasonable comparison with previous studies. Large sample numbers are required especially because some resistance patterns can be falsely highlighted if the tested number of isolates is low. Such rigorous testing for large sample numbers is especially essential in reflecting an accurate picture of the actual resistance incidence in anaerobic Gram-positive cocci where resistance rates are usually overestimated due to low isolate numbers [11,20,21,22].

From 2298 Gram-positive anaerobic isolates we see predominantly susceptible strains to penicillin with the exception of *Eggerthella lenta* (1/23) and *Lactobacillus* isolates (7/13). Although reports about susceptibility patterns of *Eggerthella lenta* are scarce, this decreased susceptibility to penicillin has been previously reported in east Germany (25%) [23], Sweden [24], and outside Europe [25,26] as in Canada (46%) [13]. Nevertheless, we see much higher resistance rates in our cohort, which may be due to differences in patient cohorts and geographic distribution of the isolates. Metronidazole is the antibiotic of choice for many clinical situations when effectivity against anaerobic bacteria is needed. In comparison with previous studies that reported decreased metronidazole susceptibility in Gram-negative bacilli, our cohort yielded high proportions of susceptibility among strains (97.3%, 472/485). On the other hand, in accordance with data published by others [23,27], Gram-positive bacteria, especially *Bifidobacterium* (33.3%) and *Atopobium* isolates (92.3%) showed the lowest susceptibility proportions. The susceptibility trends for *Atopobium, Murdochiella*, and *Bifidobacterium* species are especially interesting as they are usually underrepresented or absent in previous European studies [28,29,30]. *Veillonella* isolates showed a unique high value of MIC_90_ for Metronidazole of 16 (16/41 susceptible), which differs from previous European and international reports of a rather coherently susceptible patterns [13,23,30].

In this study, *Cutibacterium* isolates were the most common (43%) among all isolates. Most of the samples originated from neurological and orthopedic wards, which corresponds to the frequency of *Cutibacterium* infections in central nervous system (e.g., shunt infections) and prosthetic joint infections [31]. To what extent the detection of these isolates should be considered a contamination or clinically relevant could not be evaluated from the available data. The pure count may lead to overestimation of the clinical importance of this bacterium, but as most isolates originated from sterile samples, they should be considered at least potentially relevant. It is also likely that the same strains of *Cutibacterium* species cause infections as are found as contaminants. Despite the frequent occurrence of *Cutibacterium* isolates in foreign body infections, several studies showed that they remain mostly susceptible to various β-lactam antibiotics [29,32,33]. This could also be demonstrated in our isolates where over 99% of the isolates were susceptible to penicillin, the recommended first line therapy for prosthetic joint infection with *Cutibacterium* [2].

As rifampicin is a favorable combination agent in foreign body infections [16], we aimed to estimate a cutoff value for the wild-type distribution in our *Cutibacterium* cohort, which can be considered robust due to the large number of clinical isolates tested (n = 1232). Although the 99.9% ECOFF of 0.063 µg/mL cannot be automatically interpreted as a clinical breakpoint, it was substantially higher than the MIC_90_ of our isolates (0.008), which gives confidence to the estimation of our wild-type population end point. Further similar testing on a large number of isolates across different trans-national laboratories will be essential to estimate reliable ECVs. The same cannot be said about moxifloxacin, as its estimated 99.9% ECOFFs for Gram-positive and Gram-negative bacteria lies beyond the sensitivity range used here and by the CLSI. This may be due to the overlapping distributions of wild-type MIC-values of the different genera and species of Gram-positive and negative anaerobic bacteria, which complicates the identification of a specific cutoff for the wild-type populations. Genus- or species-specific breakpoints for moxifloxacin and for other antibiotics against anaerobic bacteria such as tetracyclines [34] are much needed from EUCAST to harmonize their evaluation through the different European countries.

*Bacteroides* isolates were among the most common Gram-negative anaerobic bacteria with 253 isolates mostly from drainage/dialysate samples from general surgical wards. This common occurrence underlines their potential role as a source of infection in the gastrointestinal tract where they are a commensal organism [35]. *Bacteroides fragilis* was isolated most often (52.6%) where piperacillin-tazobactam showed better activity than ampicillin-sulbactam (6.2% and 13.1% resistance, respectively), similarly to previous published studies [36,37,38]. Both *Bacteroides* isolates in general and *Bacteroides fragilis* specifically showed a good susceptibility rate to metronidazole of 95% and 98.4% (125/127), respectively, which is one of the main agents in pre-surgical prophylaxis regimes [39]. Besides moxifloxacin, clindamycin showed also low activity against *Bacteroides* populations, with only 66.4% (123/185) of strains susceptible. This is in accordance with a number of recent reports of increased clindamycin resistance among anaerobes inside and outside Europe [30,40,41,42] but even higher levels of clindamycin resistance of (49%) have been reported in Spain [11]. *Prevotella* isolates showed the highest susceptibility rate among Gram-negative bacilli against clindamycin (84.3%), beside almost full susceptibility against meropenem, metronidazole, and β-lactam/β-lactamase inhibitor combinations. This is comparable to previous European studies but higher clindamycin resistance rates have been previously reported in Canada, Kuwait, and Singapore [13,23,40].

The question of antimicrobial susceptibility data is especially relevant when urgent empirical therapies must be administrated, for instance in bloodstream infections. Blood cultures accounted for almost 16% of all tested isolates. In this group, 22% were Gram-negative with the distribution of isolates comparable with previous reports [43]. Although it can be difficult to authenticate the relevance of the presence of bacteria that are part of the normal skin microbiota such as *Cutibacterium*, the presence of certain Gram-positive and most Gram-negative anaerobes in blood cultures almost always represents true bacteremia [44]. Due to its good susceptibility rates across different anaerobic genera and also its wide aerobic activity piperacillin-tazobactam appears to be a very reasonable choice especially when it is hard to differentiate microscopically aerobic from anaerobic bacteria.

Despite the large number of isolates, this study has some limitations. Not all antibiotics were tested for all anaerobic isolates in the same group/genera; this is mostly due to the specific cases in which individual therapeutic decisions had to be made. This might have an impact on the number of isolates for specific antibiotics especially those with a low number of isolates, and this therefore may reduce the informative value. Nevertheless, our results give an overview about the current situation of antimicrobial susceptibility patterns of anaerobic bacteria in central Europe especially for some of the underrepresented anaerobes such as *Eggerthella*, and contribute valuable data that could be harnessed towards the validation of ECVs and clinical breakpoints of many antibiotics against anaerobic bacteria.

## 4. Materials and Methods

### 4.1. Isolates Collection

Non-duplicate anaerobic isolates (first clinical isolate per patient) with susceptibility testing between the years (2015–2019) in Freiburg University Medical Center have been retrospectively selected for further screening (n = 2856). Freiburg University Medical Center is a tertiary care hospital, and the Institute of Medical Microbiology and Hygiene receives around (250,000) clinical samples for microbiological analysis per year from patients mainly from Southern Germany.

### 4.2. Cultivation and Identification of Anaerobic Bacteria

Clinical materials were cultured on yeast cysteine blood agar (in-house abbreviation HCB) and incubated for 2–10 days (according to sample type) under anaerobic conditions in a jar or in plastic bags using either the Genbox ANAER (bioMérieux, Marcy-l’Étoile, France) or the Anaerocult (Merck, Darmstadt, Germany) system. Identification of anaerobic bacteria was carried out using a MALDI-TOF mass spectrometer (Bruker Daltonics, Leipzig, Germany).

### 4.3. Antimicrobial Susceptibility Testing

Susceptibility testing including determination of minimal inhibitory concentrations (MICs) was carried out by MIC test strips (Liofilchem, Piane Romano, Italy) according to the manufacturer’s instructions. Single colonies were picked from cultivated cultures and suspended in peptone broth (in-house produced) to reach a 1 McFarland. Suspended colonies were cultivated on in-house produced Brucella agar (BBA, Brucella-agar + 5% sheep blood + vitamin K) for at least 48 h under anaerobic conditions (35 ± 1 °C) before evaluating antimicrobial susceptibility. MICs were classified to susceptible, intermediate, or resistant categories according to the EUCAST guidelines for bacterial clinical breakpoints (v 10.0, released 1 January 2020) [45] for Gram-positive and Gram-negative anaerobic organisms. In most cases susceptibility to penicillin, metronidazole, moxifloxacin, and clindamycin was tested for Gram-positive anaerobes, and to ampicillin-sulbactam, meropenem, metronidazole, moxifloxacin, and clindamycin for Gram-negative anaerobes. For foreign body infections rifampicin would be additionally tested due to its biofilm activity. Susceptibility data were extracted from the laboratory information and management system [M/Lab] (DORNER Health IT Solutions, Müllheim, Germany). Anaerobic bacteria that were identified to the genus or species level but did not undergo susceptibility testing were excluded from the analysis. Furthermore, antibiotics that were tested in less than 10% of the individual bacterial isolates (genus or species) were excluded from further analysis for this specific group. MIC_50_ and MIC_90_ were calculated for bacterial groups with at least 10 isolates. In total we included 2856 unique isolates.

### 4.4. Determination of Wild-Type MIC Distributions and Cutoff Value

Assessment of MIC distribution values for various antibiotics and estimation of wild-type cut-off values for rifampicin against *Cutibacterium* were done using the ECOFFinder software as previously described [46,47] which estimates epidemiologic cut-off values based on nonlinear regression method [48]. Frequencies of MICs for each tested antibiotic were fed to the tool analyzed according to the software instructions.

### 4.5. Demographic and Clinical Data

Clinical characteristic of the patients (gender) and samples (sample type and ward) have been curated from the M/Lab program and analyzed with the R programming language (version 4.0.2). Heat-maps of isolates’ department and tissue distribution were generated using the R packages ggplot2 and pheatmap [49,50].

## 5. Conclusions

Our study provides a panoramic overview of the landscape of antimicrobial susceptibility patterns across various genera of anaerobic bacteria in a center in Southern Germany from various clinical origins. These results will improve the state of knowledge about the progress of resistant phenotypes in this underrepresented group. In summary, resistance rates of moxifloxacin and clindamycin are relatively high, emphasizing the need for antimicrobial susceptibility testing before using these antibiotics for anaerobic infections. Very low resistance rates for metronidazole and β-lactam/β-lactamase inhibitor combinations keep these antibiotics suitable for empirical therapy. For antibiotics with no defined breakpoints for anaerobic bacteria, analysis of the wild-type distribution of MICs may be used to define susceptibility locally if a sufficient number of strains are available for the analysis.

## Supplementary Materials

The following are available online at https://www.mdpi.com/2079-6382/9/11/823/s1, Figure S1: Estimation of wild-type MIC distributions for various antibiotics, Table S1: Demographic characterization of clinical anaerobe samples, Table S2: Antimicrobial susceptibility patterns of Gram-positive anaerobic bacteria, Table S3: Antimicrobial susceptibility patterns of Gram-negative anaerobic bacteria.
