# Supplementary figures and images for: Antimicrobial Susceptibility Patterns and Wild-Type MIC Distributions of Anaerobic Bacteria at a German University Hospital: A Five-Year Retrospective Study (2015–2019)
Source: Antibiotics (Basel). 2020 Nov 18;9(11):823. doi: 10.3390/antibiotics9110823 (PMC7698766; doi:10.3390/antibiotics9110823)

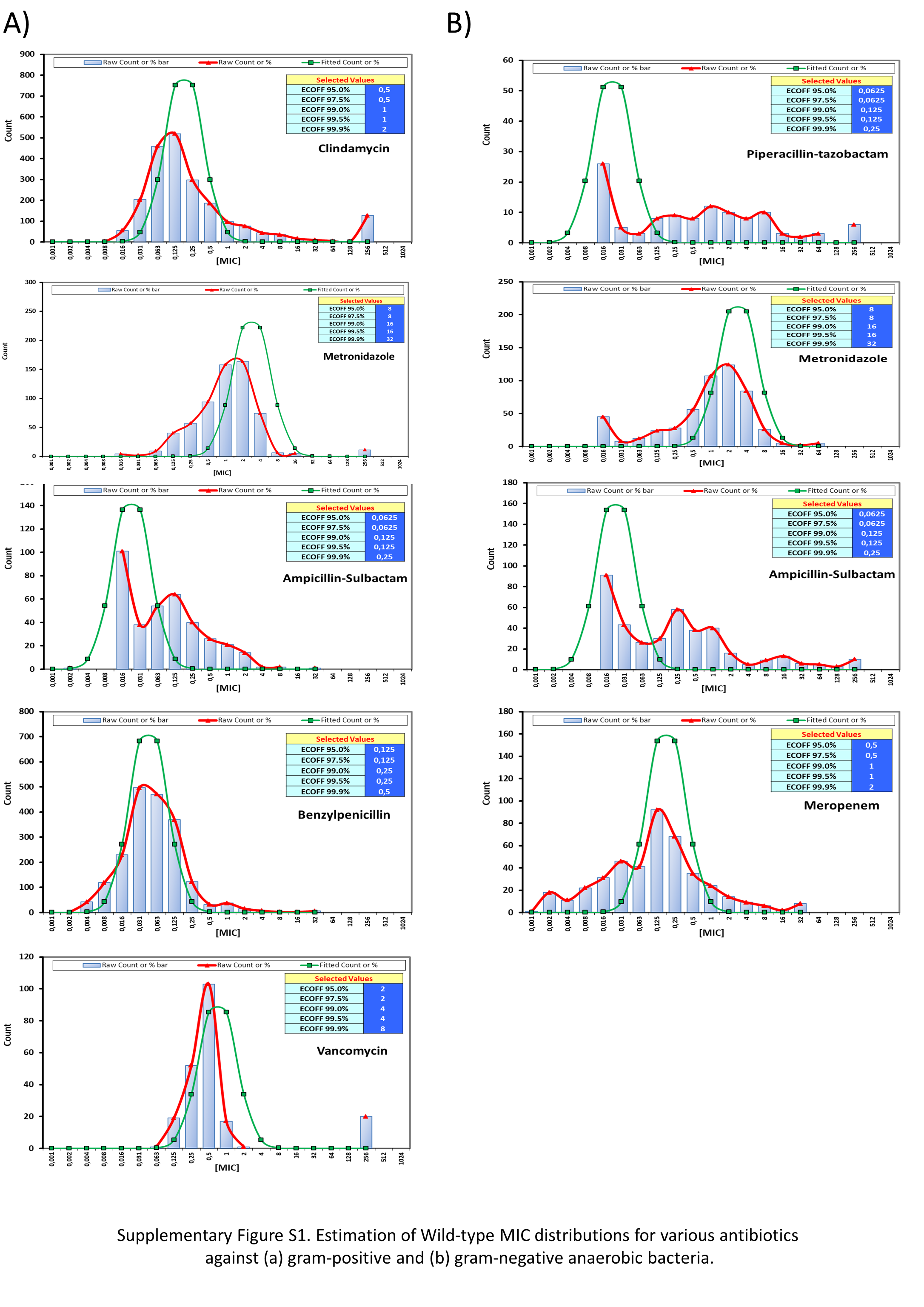

Supplement: Supplementary file 1 [file antibiotics-09-00823-s001.zip › Supplementary files/Figure_S1.tiff]
